# Digital Rehabilitation Monitoring Reveals Critical Recovery Patterns After ACL Reconstruction: A Longitudinal Analysis of 5675 Practice Data Sets in 335 Patients

**DOI:** 10.3390/jcm14196952

**Published:** 2025-10-01

**Authors:** Andreas Kopf, Wolfgang Hitzl, Christoph Bauer, Maximilian Willauschus, Johannes Rüther, Niklas Engel, Sophie Pennekamp, Lotta Hielscher, Vincent Franke, Hermann-Josef Bail, Markus Gesslein

**Affiliations:** 1 Department of Orthopaedics and Traumatology, Paracelsus Medical University, Breslauer Straße 201, 90471 Nuremberg, Germanymarkus.gesslein@klinikum-nuernberg.de (M.G.); 2 Research Office (Biostatistics), Paracelsus Medical University, Müllner Hauptstrasse 48, 5020 Salzburg, Austria; w.hitzl@salk.at; 3 Institute of Physiotherapy, School of Health Sciences, ZHAW Zurich University of Applied Sciences, 8400 Winterthur, Switzerland; 4 Lake Lucerne Institute, 6354 Vitznau, Switzerland; 5 Department of Internal Medicine 6, Paracelsus Medical University, Prof.-Ernst-Nathan-Straße 1, 90419 Nuremberg, Germany

**Keywords:** ACL reconstruction, digital rehabilitation, limb symmetry index, telerehabilitation, functional recovery

## Abstract

**Background:** Despite the high prevalence of anterior cruciate ligament (ACL) surgeries, standardized, evidence-based rehabilitation protocols remain lacking. Digital medical devices (DMDs) like the “Orthelligent” system have gained relevance as adjuncts to traditional physiotherapy, offering continuous, objective monitoring of functional recovery. **Methods:** A retrospective cohort analysis included 335 patients who underwent ACL reconstruction and used the “Orthelligent home” system between August 2022 and December 2024. In total, 5675 recorded test and exercise events were analyzed. Functional recovery was assessed using the Limb Symmetry Index (LSI) across five defined rehabilitation phases (0–4). All patients followed a structured rehabilitation program aligned with current clinical practice guidelines, supplemented by Orthelligent as a home-based digital tool for daily monitoring. **Results:** Significant functional improvement was observed during early rehabilitation phases, with the LSI increasing from 0.64 ± 0.02 in phase 0 to 0.81 ± 0.01 in phase 2 (*p* < 0.001). Time since surgery was a significant positive predictor (*p* = 0.034), while pain showed a strong negative impact on performance (*p* < 0.001). Anthropometric factors had no significant effect. Exercises associated with high rates of drop-out, pain, or difficulty were identified and linked to specific rehab phases. **Conclusions:** This study demonstrates that digital rehabilitation monitoring can reliably reflect patient progress after ACL reconstruction. The early postoperative period (first 3 months) is critical for functional gains, highlighting the need for individualized, pain-sensitive rehabilitation strategies.

## 1. Introduction

Joint-preserving arthroscopic and open surgical operations are one of the most frequently performed orthopaedic surgical procedures [1]. Nevertheless, there are no uniform post-treatment recommendations regarding the rehabilitation process [2,3]. These vary almost from institution to institution. Recently, there have been attempts to improve this through scientific studies. However, most such studies relate to ACL-reconstruction interventions and only a few to other pathologies [2,4,5]. However, the studies all agree that good rehabilitation plays a decisive role in the patient’s outcome [6].

ACL injuries are among the most common sports- and trauma-related knee injuries, with an estimated incidence of 68.6 per 100,000 person-years in young athletes [7]. Non-contact mechanisms such as sudden pivoting, deceleration, or valgus stress ac-count for the majority of ACL ruptures, while contact injuries occur less frequently [8]. Surgical reconstruction is typically performed using autografts (e.g., hamstring or patellar tendon) or allografts, with arthroscopic techniques being the current standard of care [9]. Despite high surgical success rates, postoperative outcomes strongly depend on rehabilitation quality [2]. These considerations highlight the need for standardized rehabilitation pathways and justify the evaluation of digital monitoring systems like Orthelligent as potential tools to improve recovery and adherence.

Over the last few years, a trend has emerged in the study situation away from the classic rehabilitation approach with physiotherapy alone towards supplementary interventions using digital medical devices [10]. Devices such as the Orthelligent tool from OPED (Valley, Germany) are usually not used on their own but are designed to supplement rehabilitation sports and physiotherapy for use at home [10]. In the course of this development, a number of studies were also carried out to evaluate whether measurement using individual sensors is adequate and reliable [11].

Recent studies have increasingly investigated the role of digital and virtual rehabilitation modalities after ACL reconstruction. A recent meta-analysis demonstrated that virtual reality-based rehabilitation significantly improves knee function, strength, and balance compared to standard care [12]. Another systematic review confirmed that VR-based interventions reduce pain and enhance dynamic stability [13]. Moreover, mobile health–based home rehabilitation programs have been shown in randomized trials to improve early outcomes and patient compliance [14]. These data underline the contemporary relevance of integrating digital solutions such as Orthelligent into standardized ACL rehabilitation pathways.

Some studies have also been able to demonstrate effectiveness in terms of an improved rehabilitation process. This was shown by increased adherence to the rehabilitation protocols and an increased willingness to exercise compared to patients without the support of such tools [10,15,16,17].

Although there are data for “return to play” periods from professional football, it remains unclear how long normal patients need on average to be able to move without pain after knee surgery [18]. It is also unclear to what extent recommendations for postoperative weight-bearing and exercise as well as exercises for the knee can be applied [2].

For this reason, this study aimed to further evaluate how an average rehabilitation process can be structured and in which rehabilitation phases the most functional progress is achieved. The secondary aim is to identify which parameters show particular influence on the rehabilitation progress, such as anthropometric properties or pain, which is known to increase the risk of postoperative arthrofibrosis. Finally, we aimed to determine which rehabilitation exercises or groups of exercises show poorer results and should therefore be adapted in the rehabilitation process.

## 2. Materials and Methods

### 2.1. Study Design and Population

In a retrospective cohort analysis, routine data of patients following anterior cruciate ligament reconstruction (ACLR) were analyzed, which were documented using the digital medical product “Orthelligent”. The data corresponds to the routine data of the sensor and were collected anonymously in the period from August 2022 to December 2024. The study population therefore includes all patients who used the “Orthelligent home” version in the defined period. As the Orthelligent system is distributed throughout Germany, included patients underwent ACL reconstruction at different orthopedic centers across the country. Surgeries were not limited to a single institution, and postoperative rehabilitation was carried out either at local physiotherapy practices or at home using the Orthelligent home system. After knee surgery, patients are prescribed the app by their orthopaedic surgeon as an additional tool to normal rehabilitation. It was also possible to acquire the app independently and without a prescription by borrowing the sensor for a fee. Baseline demographic data (sex, age, BMI, height, weight) were collected and are summarized in Table 1. Information on concomitant pharmacological treatments (e.g., analgesics and thromboprophylaxis) and perioperative complications was not systematically available in the dataset and is therefore acknowledged as a limitation.

The study was also registered with the research management of Paracelsus Medical University (FMS_IF_006.25-I-2; Regeneration) and it was evaluated by the Institutional Review Board (IRB-2025-08) that no ethics vote is necessary.

AI Statement: Artificial intelligence (AI) tools were used to support the drafting and editing of this text. All final decisions on content, wording, and interpretation were made by the authors.

### 2.2. Inclusion and Exclusion Criteria

**Inclusion criteria**: Time of surgery within this period and at least one test. The indication in the sense of an isolated or combined injury to the anterior cruciate ligament was also an inclusion criterion. The indication in terms of an isolated or combined injury to the anterior cruciate ligament was also an inclusion criterion. This resulted in 4 injury patterns that could be included: (1) Isolated ACL rupture; (2) ACL rupture and meniscus injury; (3) ACL rupture and collateral ligament injury; (4) ACL rupture with collateral ligament and meniscus injury. Aborted tests were also documented by the sensor, and the app subsequently asked why the test was aborted so that these tests could also be included in the evaluation and an assessment of the difficulty of the exercises could be carried out.

**Exclusion criteria**: All those who did not have a corresponding indication or surgery in the defined time were excluded. All Patients older than 65 years were also excluded.

### 2.3. Structure of the Rehab Program

The rehabilitation program, which forms the basis for the algorithm, consists of five phases based on established ACL clinical practice guidelines [2,3]. At the beginning, each patient was asked to state the type of injury, the treatment that had taken place or was about to take place, as well as anthropometric data such as height and weight. Each patient could choose training frequency per week and change this setting during progress. The patient was asked to indicate whether they are in a pre- or post-operative phase and provide a surgery date. The age was recorded in age groups from 0 to 65 years. From 0 to 15 and from the age of 15 in steps of five years.

An initial evaluation test was then carried out on the healthy leg and the injured leg, in which the range of motion, flexion and extension, and stability in the single-leg stance were tested. These tests calculated the flexion in degrees and the extension deficit in millimetres, as well as the limb symmetry index from the comparative data with the healthy leg.

Each patient started in rehabilitation phase 0. After the evaluation test, the patient was assigned exercises, depending on the preset exercise frequency, which they had to complete with the sensor attached and then indicate the difficulty and potential pain in the app. The exercises were assigned to the rehabilitation phases and evaluated by previous studies and a panel of OPED experts so that patients are not given exercises that are too difficult at too early a stage [10].

Exercise content ranged from basic range-of-motion and weight-bearing activities in early phases to strength, neuromuscular control, and plyometric training in later phases. Patients reported training frequency (number of sessions per week) individually, as mentioned, and the Orthelligent app adapted exercise difficulty accordingly. This ensured gradual, guideline-based progression while allowing individualized adjustments.

**Figure 1:**
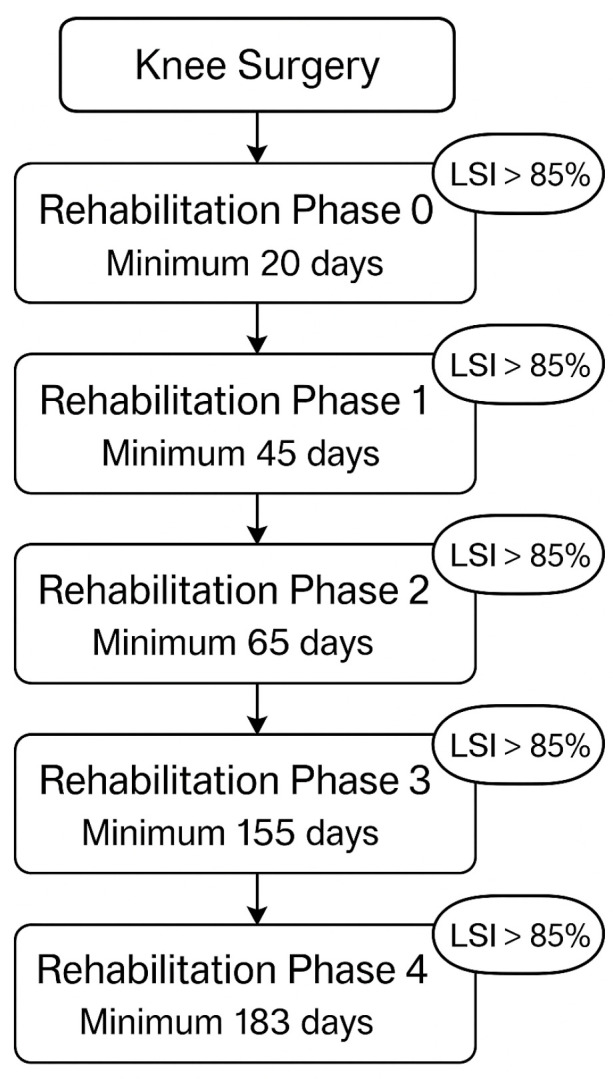
Flow chart of the rehabilitation progress.

After completing an exercise phase for a defined period, a new evaluation test was carried out followed by a new assignment to the rehabilitation phases. Progress can only be made if the LSI is greater than 85% and a minimum number of days have been completed. Phase 0 had to be completed after at least the 20 postoperative day, phase 1 by day 45, phase 2 by day 65, phase 3 by day 155, and phase 4 by day 183 (Figure 1). This prevents too rapid progression and possible overloading and damage.

Depending on the progression of the rehabilitation process, the exercises became more difficult, coordinatively more complex and also require more strength (e.g., Phase 0: Seated Single Leg Quad Extension, Leg-lift-supported; Phase 1: Single Leg Balance, Mini-squat; Phase 2: Copenhagen Plank Dynamic, Side Lying Adductor Raise; Phase 3: Sumo Squat, Elevated Hamstring Plank–Dynamic; Phase 4: Lying-squat, Single Leg Glute Bridge on Wall). Different levels and units were measured for each test, so the exercises could be compared with each other. As the LSI was recorded for all exercises, a comparison of the rehabilitation progress over time was possible. A drop-out was defined as not achieving or breaking off the exercise goal set, in particular when a patient stopped the exercise, and the patient was asked directly by the app for the reason of abortion.

### 2.4. Statistical Analysis

IBM SPSS Statistics Version 30.0, Microsoft Excel (Microsoft 365) and Cloud Software Group, Inc. (2023) Data Science Workbench, Version 14, were used for the statistical analysis. Generalized linear models (GLM) with generalized estimating equations (GEE) were used to quantify functional developments over the course of rehabilitation. This method allows a robust estimation of correlated longitudinal data, as they are generated in the context of repetitive testing over different rehabilitation phases.

The dependent variable was the limb symmetry index (LSI), measured in percent, with a normally distributed error structure and identity link function. The independent variables included elapsed time since surgery and covariates such as pain score, body weight and height. The rehabilitation phase was included as a categorical influencing factor. The Wald-Chi^2^ test was used to test the significance of individual influencing variables.

Supplementary analyses were performed, including pairwise comparisons of estimated marginal means between rehabilitation phases, as well as the influence of categorical predictors on pain data using Chi^2^ tests. In total, the analysis comprised 5675 individual measurements in 335 patients. The focus was on identifying the rehabilitation phases with the greatest functional progress and determining the factors that significantly influence these developments. The results provided differentiated findings on the effectiveness of digitally supported rehabilitation processes and their controllability through relevant clinical and subjective parameters.

## 3. Results

### 3.1. Patient Demographics

A total of 5675 test and exercise events of 335 patients after anterior cruciate ligament (ACL) reconstruction were retrospectively analyzed. The mean number of test repetitions per patient was 17 (minimum = 1, maximum = 164). The assignment to a patient was represented by an individual ID, whereby a wide range of rehabilitation phases and exercise intensities were documented. The majority of patients were in the early rehabilitation phases (phase 0–2), with significant functional increase over time. The anthropometric data of this population according to injury patterns are shown in Table 1.

### 3.2. Functional Development

**Figure 2:**
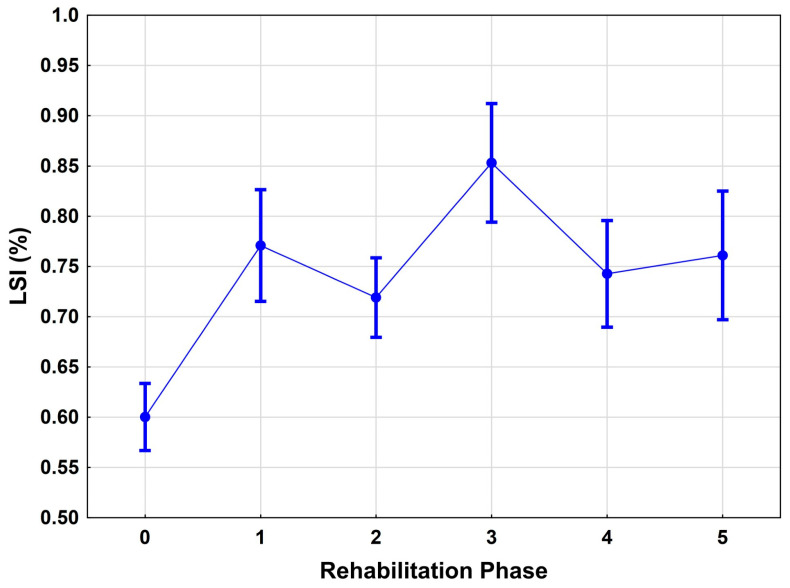
Effects of the rehab phases over time.

The analysis of the functional results using the Lim Symmetry Index showed continuous improvement over the course of the rehabilitation period. Between phases 0 and 2, the mean LSI score increased significantly from 0.64 ± 0.02 to 0.81 ± 0.01 (*p* < 0.001) (Figure 2). The elapsed time since surgery proved to be a significant positive predictor of functional development (*p* = 0.034). Conversely, there was a strong negative effect of pain (measured on the VAS) on performance (*p* < 0.001). Height and weight had no significant effect on the test results (Table 2).

**Figure 3:**
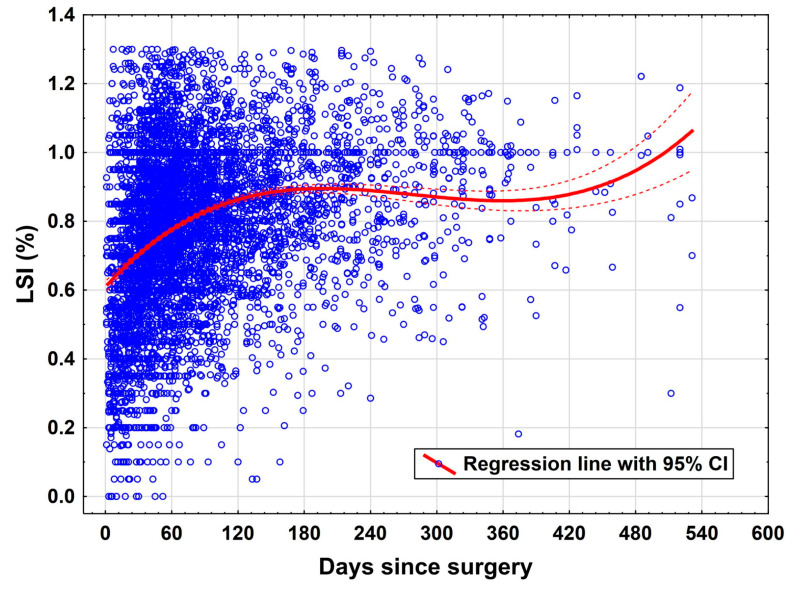
Rehab progress measured by the LSI (FIT) over the time.

**Figure 4:**
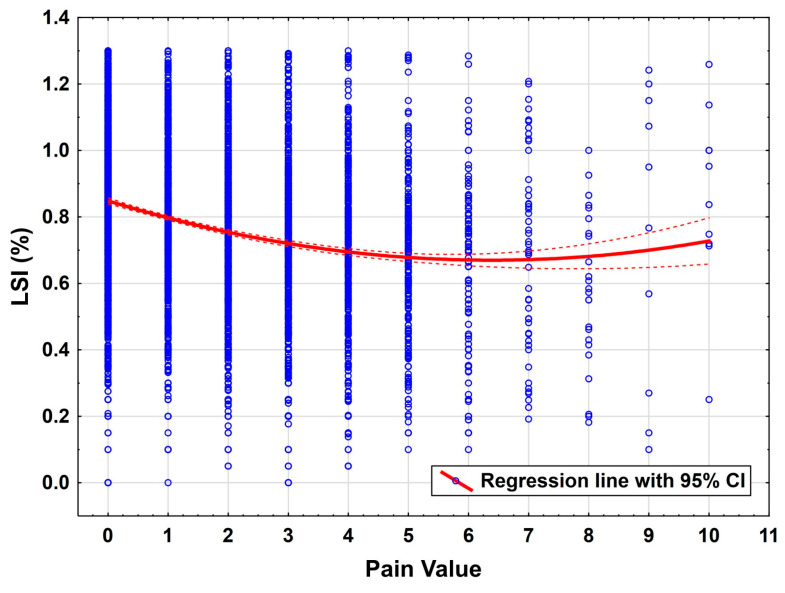
Rehab progress measured by the LSI (FIT) against the pain while performing.

There was a significant influence of time on the functional values (Figure 3) and the pain level during the exercises (Figure 4). Here, the rapid improvement in the early rehab phases in the first 150 days can be visualized particularly well, as well as the negative correlation of the pain value also in the early rehab phases. Figure 4 illustrates the progressive decline in pain intensity with higher LSI, with the steepest reduction occurring in the early rehab phases. Clinically, this emphasizes the critical importance of early pain management strategies to facilitate functional recovery.

### 3.3. Exercise-Specific Outcomes

**Figure 5:**
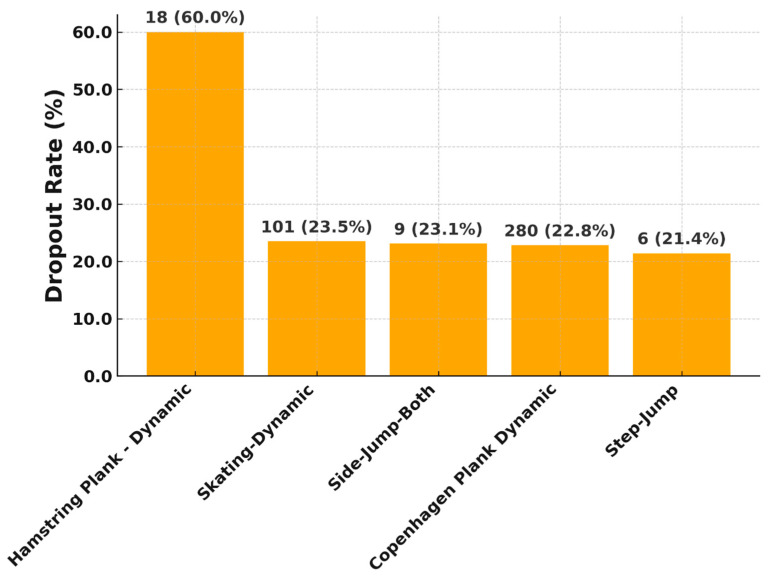
Top 5 exercises with the highest drop out.

The top five exercises with the highest drop-out rates are demonstrated in Figure 5. Different absolute frequencies result from the fact that the exercises are tested with different frequencies and according to the algorithm in different rehab phases. Figure 5 shows dropout rates per exercise type, with high attrition observed in all these types of complex exercises. This indicates that some advanced tasks may require modification or gradual introduction to avoid patient demotivation.

**Figure 6:**
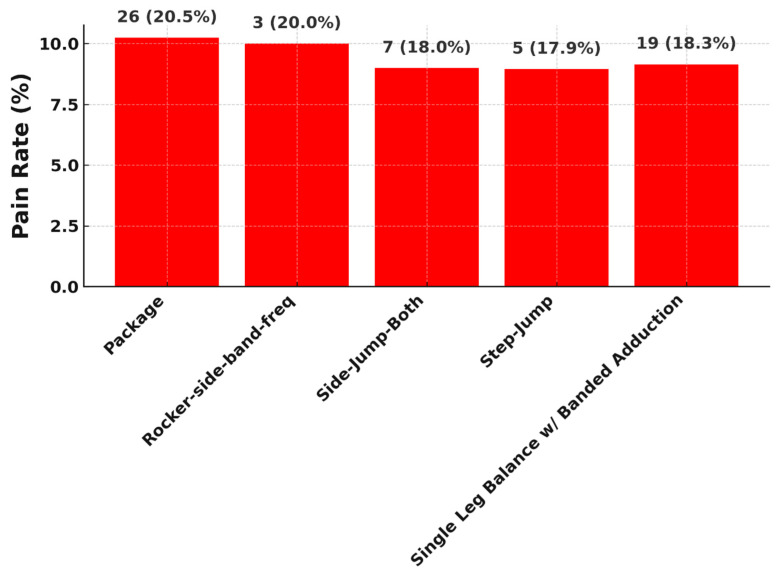
Top 5 exercises with the highest pain values.

In addition, five exercises were identified with the highest pain values (each around 20%) are demonstrated in Figure 6.

**Figure 7:**
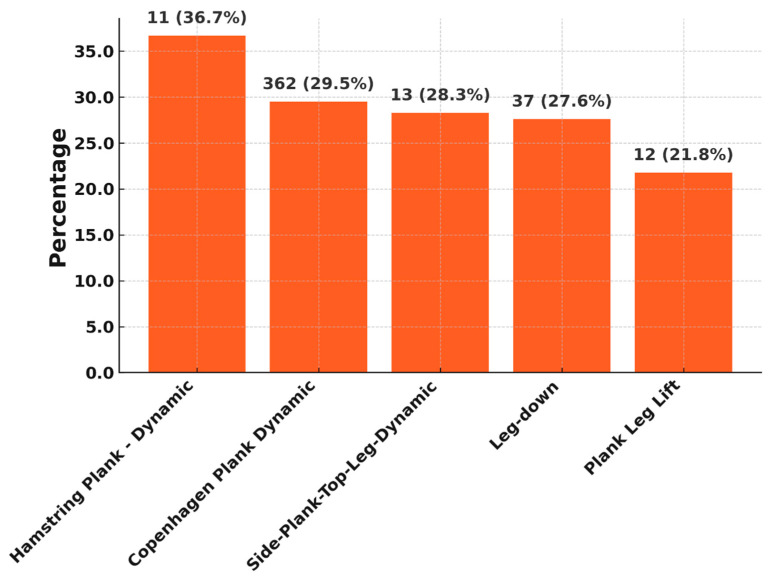
Top 5 exercises with the worst feedback named “too hard”.

Finally, the top five exercises with the most frequent “too hard feedback” are listed in Figure 7.

Overall, there was a clear decrease in these three categories over the rehabilitation phases 0–5 (too hard: 7.5% vs. 2.9%; pain during exercise: 4.4% vs. 1.6%; drop-out rate: 8.0% vs. 5.5%).

## 4. Discussion

The most important finding of this study was that the use of an app-structured rehabilitation program showed significant functional improvement during early rehabilitation phases. Time since surgery was a significant positive predictor while pain showed a strong negative impact on performance.

A common problem in rehabilitation research to date—particularly in the orthopaedic–surgical context—is that there are hardly any robust datasets available that map the time course of functional recovery in detail. The vast majority of studies are limited to selective measurements (e.g., 6 or 12 weeks postoperatively) or only record final outcome values (e.g., return to sport after 6 months) [4,18]. In this respect, our present data collection with thousands of feedback on a variety of exercises and classification according to the rehabilitation phases (0–5) could be useful for further development of artificial intelligence (AI)-based rehabilitation programs. For the first time, it allows a phase-related analysis for increased pain or problems during exercising. Exercises that lead to dropouts in certain rehabilitation phases were identified.

Systematic longitudinal reported data on exercise adherence, resilience, and pain changes over the course of time are urgently needed for shaping a personalized rehabilitation approach for each individual patient. This study based on routine data is unique to date and fills a key gap in the literature.

Nevertheless, classification and interpretation of the data must be assessed carefully. By using routine data from a system that potentially improves the rehabilitation process, it is unclear whether these results can be transferred to the general population. Studies such as that by Höher et al. 2023, Essery et al. 2017 or Dunphy et al. 2020 showed increased adherence and patient satisfaction when using digital systems such as Orthelligent [10,16,17]. The functional added value compared to standard procedures remains controversial. Randomized controlled trials with robust functional endpoints are lacking.

Digital monitoring can create valuable data, which can be used to investigate the microdynamic patterns of the rehabilitation process and thus still provide reliable results due to the large heterogeneous population. The documented reduction in pain and drop-outs, combined with increasing exercise quality (e.g., more “perfect” feedback), allows valid conclusions to be drawn about typical recovery patterns.

### 4.1. Functional Recovery Patterns

The present analysis demonstrates a clear, statistically significant improvement in functional capacity as measured by the Limb Symmetry Index (LSI) over the course of rehabilitation. Specifically, the LSI increased from a mean of 0.64 in phase 0 to 0.81 in phase 2, reflecting an early and marked gain in lower extremity function (*p* < 0.001). These results confirm previous findings that the most substantial functional improvements typically occur in the early post-operative period, often within the first 3 to 6 months after anterior cruciate ligament reconstruction (ACLR) [4,19].

Our results align with recent evidence on digital rehabilitation approaches. Virtual reality interventions have been reported to enhance functional outcomes and reduce pain in ACL patients [12,13]. Similarly, mobile health applications have been shown to improve early adherence and recovery [14]. In contrast to these studies, our work provides a longitudinal analysis of over 5000 test events, capturing recovery trajectories across five rehabilitation phases. This design highlights the originality of our contribution and complements existing data from smaller, highly controlled trials.

The most prominent predictor of functional improvement in this study was time elapsed since surgery. The variable “days since surgery” was associated with a statistically significant positive effect on LSI (*p* = 0.034). This aligns with longitudinal rehabilitation studies that indicate a gradual, time-dependent recovery of neuromuscular control and muscular symmetry post ACLR [4,19,20]. However, it is important to recognize that this temporal association does not necessarily reflect a uniform progression across individuals, as adherence to rehabilitation protocols and individual recovery rates vary widely.

### 4.2. Pain as a Limiting Factor

Pain as assessed by the Visual Analogue Scale (VAS) was found to have a strong negative influence on performance (*p* < 0.001). This emphasizes the central role of pain management in functional rehabilitation. Pain can act as both a mechanical and psychological inhibitor of motor performance, which in turn may suppress engagement in critical neuromuscular training components and is known as a risk factor for arthrofibrosis [21,22]. Therefore, early and sustained pain control should be considered a priority within structured rehabilitation programs.

### 4.3. Anthropometric Factors

Contrary to some previous assumptions, anthropometric parameters such as body height and body weight showed no significant effect on functional outcomes in this cohort. While several biomechanical models suggest that increased body mass may impose greater joint loading and potentially delay recovery [23,24,25], this was not supported in the present data. The lack of effect may reflect the fact that standardized load-adapted rehabilitation strategies (such as those implemented by “Orthelligent”) are able to compensate for individual anthropometric differences.

### 4.4. Exercise-Specific Challenges

Certain exercises showed a high proportion of “too hard” ratings from patients. This indicates a perceived high subjective load, which must be viewed critically in the early phase of rehabilitation. The Hamstring Plank-Dynamic exercise, with over 36% “too hard” feedback, is the most stressful. This is in line with findings from the literature, which show that hamstring-dominant exercises are biomechanically and proprioceptively challenging in early postoperative phases and require a high level of muscular control [2].

Copenhagen planks are also considered high intensity in the sports physiotherapy literature and are typically used in prevention and late rehabilitation. Their early use can lead to excessive demands, especially without sufficient supervision [15]. The fact that these exercises in the algorithm-controlled sensor are already used in late rehabilitation phases and that the total number of exercises that are too difficult decreases significantly over the rehabilitation phases indicates that there tends to be a small group of patients who need a little longer for such difficult exercises.

All of the listed exercises involve multi-joint, load-bearing movements with either dynamic instability or unilateral loading, which are known to provoke higher neuromuscular demand. Exercises like the Step Jump and Side Jump Both involve rapid plyometric motion and sudden load deceleration, which can aggravate discomfort particularly in patients recovering from anterior cruciate ligament reconstruction (ACLR) or other post-surgical knee states. Prior studies indicate that jump-landing tasks are frequently associated with elevated joint forces and increased muscle co-contraction around the knee, both of which contribute to higher pain perception during rehabilitation [26,27]. The Single Leg Balance with Banded Adduction, despite its apparent simplicity, requires deep hip and core activation while challenging proprioception and stability. The use of elastic resistance bands intensifies the adduction load and can strain recovering tissues, especially in patients with deficits in gluteal strength or neuromuscular control [28]. Similarly, Rocker Side Band-Frequent is characterized by repetitive lateral movement with band resistance, stressing the lateral kinetic chain, which can provoke discomfort due to the constant tension and low-level isometric hold requirements. Exercises like Package-which may involve a compound or novel movement pattern-are often rated painful because they lack individualized progression schemes and may not be well aligned with patients’ current functional levels. Evidence suggests that poorly timed progression or lack of personalization in rehabilitation programming increases the likelihood of overexertion and pain flare-ups [29].

### 4.5. Clinical Implications

The present data offer, for the first time, a differentiated view of functional development across defined rehabilitation phases based on a large volume of real-world data. This is particularly valuable for identifying time windows for targeted interventions, adapting rehab progressions, and personalizing therapy plans. While causal interpretations remain limited due to the observational nature of the data, the findings strongly suggest that routine DMD-generated data can validly reflect recovery progress and contributing factors, especially in early rehab stages. In clinical practice, digital rehabilitation monitoring may support therapists and surgeons by providing objective feedback on patient progress, enabling early detection of pain-related limitations, and facilitating individualized exercise progression. These insights can be readily implemented to optimize patient management in the early postoperative phase. However, barriers to widespread adoption include costs of digital systems, variability in acceptance among clinicians, and the need for integration into existing healthcare infrastructures. Addressing these issues will be essential for broader implementation.

### 4.6. Limitations and Strengths

This study presents unique insights into functional rehabilitation progress using routine data collected via a digital medical device (DMD). Nevertheless, several limitations must be acknowledged.

**Limitations**: First, the retrospective, observational design inherently limits causal inferences. Although generalized estimating equations (GEE) were employed to account for intra-individual correlation across repeated measurements, unmeasured confounders and individual variability (e.g., in adherence, preoperative condition, psychological readiness) may still bias the results. Second, reliance on self-administered sensor-based exercises may introduce measurement variability. Although studies have validated the reliability of such systems, the consistency of execution without direct clinical supervision can vary, particularly in patients with low motor control or comprehension of instructions. Third, while the pain, “too hard,” and dropout rates offer novel insights, these remain subjective and may be influenced by individual thresholds, motivation, or environmental factors. Furthermore, a key limitation of our study is the absence of direct quadriceps morphology and strength assessment. Quadriceps recovery is a central determinant of functional outcome after ACL reconstruction and is strongly linked to return-to-sport rates and prevention of re-injury. Because the Orthelligent system does not include standardized force testing, quadriceps data could not be incorporated. Future studies should combine digital monitoring with objective dynamometric strength measurements to provide a more comprehensive picture of rehabilitation progression. Additionally, information on concomitant treatments (e.g., medications) and potential complications were not obtained by the application system and are therefore not taken into account. Furthermore, while the present analysis benefits from the large sample size and real-world character of the data, generalizability is limited. Patients were treated in diverse centers across Germany, and rehabilitation strategies were heterogeneous. Thus, the results may not fully translate to healthcare systems with different structures, reimbursement schemes, or cultural factors. At the same time, this heterogeneity mirrors actual routine care, which can be regarded as a strength, since it enhances the ecological validity of the findings.

**Strengths**: However, the study also possesses several notable strengths. With over 5.600 individual test events across 335 patients, the dataset represents one of the largest collections of rehabilitation progress post-ACL reconstruction. Unlike highly controlled RCTs that may lack generalizability, this dataset reflects routine care patterns across a heterogeneous population. Unlike most prior studies that rely on snapshots at fixed timepoints (e.g., 6 or 12 weeks post-op), this analysis captures functional change continuously across distinct rehabilitation phases.

This allows, for the first time, an evidence-based assessment of when peak functional gains occur and how patient-reported burden (pain, difficulty) evolves over time. By distinguishing between early (phases 0–2) and later rehabilitation stages, the study identifies a “therapeutic window” for maximal improvement, supporting prior findings of nonlinear recovery trajectories.

## 5. Conclusions

This study presents a novel longitudinal analysis of functional recovery based on data from a digital rehabilitation system. The findings highlight the critical importance of the early post-operative period (first 3 months) for functional gains and emphasize the need for individualized, pain-sensitive rehabilitation strategies. Further investigation using digital medical devices in combination with a digital rehabilitation app is needed to improve individual pain-free support and the personalized rehabilitation approach.

Digital medical devices provide not only personalized support but also valuable insights to optimize and structure future rehabilitation pathways. The identification of exercises with high dropout rates, pain levels, and perceived difficulty allows for more targeted exercise prescription and timing within rehabilitation protocols.

## Figures and Tables

**Table 1:** Anthropometric data and injury patterns of the population.

Group	ACL Injury	ACL + Meniscus Injury	ACL + Meniscus + Collateral Ligament Injury	ACL + Collateral Ligament Injury
**Height (cm)**	172.4 ± 8.7	177.9 ± 12.3	175.9 ± 8.8	172.0 ± 8.3
**Weight (kg)**	73.4 ± 14.7	75.4 ± 14.8	79.0 ± 14.6	71.5 ± 14.7
**BMI (kg/m^2^)**	24.7 ± 5.0	23.8 ± 4.7	25.5 ± 4.7	24.2 ± 5.0
**Age 0–15 in *n* (%)**	3 (0.9%)	0 (0.0%)	1 (0.3%)	0 (0.0%)
**Age 16–20: *n* (%)**	14 (4.8%)	27 (8.1%)	9 (2.7%)	1 (0.3%)
**Age 21–25: *n* (%)**	30 (9.0%)	24 (7.2%)	8 (2.4%)	5 (1.5%)
**Age 26–30: *n* (%)**	25 (7.5%)	20 (6.0%)	10 (3.0%)	2 (0.6%)
**Age 31–35: *n* (%)**	14 (4.8%)	16 (4.8%)	4 (1.2%)	2 (0.6%)
**Age 36–40: *n* (%)**	16 (4.8%)	10 (3.0%)	8 (2.4%)	5 (1.5%)
**Age 41–45: *n* (%)**	30 (9.0%)	10 (3.0%)	11 (3.3%)	2 (0.6%)
**Age 46–50: *n* (%)**	4 (1.2%)	7 (2.1%)	5 (1.5%)	2 (0.6%)
**Age 51–55: *n* (%)**	3 (0.9%)	5 (1.5%)	2 (0.6%)	1 (0.3%)
**Age 56–60: *n* (%)**	1 (0.3%)	2 (0.6%)	2 (0.6%)	2 (0.6%)
**Age 61–65: *n* (%)**	0 (0.0%)	0 (0.0%)	1 (0.3%)	0 (0.0%)
**Sex distribution (F/M) (%)**	F = 46.4/M = 53.6	F = 40.5/M = 59.5	F = 45.9/M = 54.1	F = 45.5/M = 54.5

**Table 2:** Influencing factors that significantly affect functional rehabilitation outcomes measured by the LSI in bold.

Predictor	*p*-Value
**Rehab phase**	<0.001
**Days since surgery**	0.034
**Body height**	0.775
**Body weight**	0.232
**Pain level (VAS score)**	<0.001

## Data Availability

The data generated in this study are included in the results of the published article.
